# Raltitrexed increases radiation sensitivity of esophageal squamous carcinoma cells

**DOI:** 10.1186/s12935-019-0752-y

**Published:** 2019-02-18

**Authors:** Wen-Xiu Ding, Shu Liu, Jian-Xin Ma, Juan Pu, Hai-Jing Wang, Shu Zhang, Xin-chen Sun

**Affiliations:** 1 Department of Radiation Oncology, The First Affiliated Hospital of Nanjing Medical University, 300 Guanzhou Road, Nanjing, 210029 Jiangsu China; 2 Department of Radiation Oncology, The Sixth Affiliated Hospital of Yangzhou University, Taixing Peoples’ Hospital, Taizhou, Jiangsu China; 3 Department of Radiation Oncology, Lianyungang Oriental Hospital, Lianyungang, Jiangsu China; 4 Department of Radiation Oncology, Lianshui Peoples’ Hospital, Huaian, Jiangsu China; 5 Department of Radiation Oncology, The Second Affiliated Hospital of Nanjing Medical University, Nanjing, China

**Keywords:** Raltitrexed, Esophageal cancer, Radiosensitization, Cell cycle arrest, Cell apoptosis

## Abstract

**Background:**

Radiation therapy remains an important therapeutic modality, especially for those patients who are not candidates for radical resection. Many strategies have been developed to increase the radiosensitivity of esophageal cancer, with some success.

**Methods:**

This study was conducted to determine whether raltitrexed can enhance radiosensitivity of esophageal squamous cell carcinoma (ESCC). ESCC cell lines 24 h were incubated with raltitrexed or DMSO with or without subsequent irradiation. Cell Counting Kit assay-8 assay and clonogenic survival assay were used to measure the cell proliferation and radiosensitization, respectively. Flow cytometry was utilized to examine cell apoptosis and cell cycle distribution in different groups. Immunofluorescence analysis was performed to detect deoxyribonucleic acid (DNA) double-strand breaks. In addition, the expression levels of proteins that are involved in radiation induced signal transduction including Bax, Cyclin B1, Cdc2/pCdc2, and Cdc25C/pCdc25C were examined by western blot analysis.

**Results:**

The results indicated that raltitrexed enhanced radiosensitivity of ESCC cells with increased DNA double-strand breaks, the G2/M arrest, and the apoptosis of ESCC cells induced by radiation. The sensitization enhancement ratio of 1.23–2.10 was detected for ESCC cells with raltitrexed treatment in TE-13 cell line. In vitro, raltitrexed also increased the therapeutic effect of radiation in nude mice.

**Conclusion:**

Raltitrexed increases the radiosensitivity of ESCC. This antimetabolite drug is promising for future clinical trials with concurrent radiation in esophageal cancer.

## Background

Nowadays, esophageal cancer has been identified as the sixth most common cause of cancer-related death all over the world, and more than one third of esophageal cases are diagnosed at advanced stage [1]. Esophageal squamous cell carcinoma (ESCC) accounts for more than 90% of this cancer in China [2]. Although there are three major treatment modalities for esophageal cancer including surgery, radiotherapy and chemotherapy, radiotherapy is widely applied to the patients who are not candidates for operations [3–5]. However, intrinsic radioresistance is developed in large portion of cancer cells of esophageal carcinoma patients, resulting in the failure of treatment, therefore, it is urgent to develop methods to increase the radiation sensitivity to reduce the mortality of esophageal cancer.

Raltitrexed, also named ZD1694 or Tomudex, has been considered as a specific and long-lasting inhibitor against thymidylate synthase (TS). As we know, TS plays a critical role in DNA synthesis and repair. Several clinical studies have indicated that TS is increased in tumor tissues, suggesting TS is involved in tumorigenesis or progression. Raltitrexed, as an TS inhibitor, is well known to be effective in the treatment of colorectal cancer. Meanwhile, preclinical studies reported that raltitrexed also showed antitumor effect in other kinds of cancers due to its direct anti-proliferation and anti-apoptosis effects, including colorectal cancer, malignant mesothelioma, head and neck cancer, hepatic cancer and gastric cancer [6–11]. Raltitrexed has also been considered as an antimetabolic drug, which could induce DNA damage [12] and cell cycle arrest [13]. However, there is little known about the effect of raltitrexed in improving the radiosensitivity of ESCC and according molecular mechanism. In the present study, we investigated raltitrexed-mediated radiosensitization and the underlying molecular mechanism.

## Materials and methods

### Reagents, cell culture, and irradiation

Raltitrexed, generously provided by Jiangsu Zhengda Tianqing Pharmaceutical Co., Ltd. (Nanjing, China), was dissolved in dimethyl sulfoxide (DMSO, Sigma, St. Louis, USA) as a stock concentration of 1 mol/l. Two human esophageal cancer cell lines, Kyse150 and TE-13, were obtained from Shanghai institute of Biological Science, China. Cells were cultured in RPMI 1640 (Hyclone, Thermo Scientific, MA) supplemented with 10% fetal bovine serum (Hyclone), 100 U/ml penicillin and 100 mg/l streptomycin at 37 °C in a humidified atmosphere of 5% CO_2_. Irradiation was delivered at a dose rate of 566 cGy/min, using an X-ray medical linear accelerator (Elekta Precise, Sweden). Cells were irradiated at a single dose at room temperature.

### Cell counting kit 8 assay

The cytotoxicity effect of raltitrexed was measured by Cell counting kit 8 (CCK-8) assay (Beyotime Technology, Shanghai, China). Briefly, cells were plated in 96-well plates at a density of 4 × 10^3^ cells/well. Then, fresh medium was added with or without raltitrexed after 24, 48, and 72 h incubation. Following the treatment, CCK-8 reagent (10 μl/well) was added into each well and cells were incubated with CCK-8 reagent for 1 h. The absorbance ratio of each sample was measured at 450 nm using a microplateReader (BioTek Elx800, Winooski, VT, USA). All experiments were repeated at least three times.

### Cell proliferation assay after radiation

For combination treatment, TE-13 and Kyse150 cells were plated at a concentration of 6 × 10^3^ cells/well in 96-well plates for18 h. Then cells were treated with raltitrexed (4 nmol/l [nM]) for 24 h and different radiation doses (0, 2, 4, 6, 8 Gy). 48 h later, CCK-8 assay Kit was used to assess cell proliferation. The OD ratio was used to calculate the cell proliferation capacity. All experiments were repeated three times.

### Clonogenic survival assay

Cells were seeded at varying cell densities in six-well plates after trypsinization, which were treated with raltitrexed (4 or 8 nM) or DMSO as control for 24 h, and then subjected to X-rays of 0, 2, 4, 6, 8 Gy. The cells were cultured at 37 °C for another 10 days, following fixing with 75% ethanol for 15 min and then staining with crystal violet for 10 min. The colonies with more than 50 cells were counted. The mean inactivation dose in raltitrexed treated cells divided the mean inactivation dose in control cells as the survival enhancement ratio (SER) that combined with single-hit multi-target model to make cell survival curves. All experiments were repeated three times.

### Cell cycle analysis

TE-13 and Kyse150 cells were incubated in six-well plates (2 × 10^5^ per well) in RPMI-1640 medium without serum for 18 h and divided into the four groups, respectively: control, raltitrexed (4 nM), irradiation (4 Gy), and irradiation with raltitrexed (4 Gy + 4 nM). Each group was repeated in three wells. After a 24 h exposure to raltitrexed followed by a 24 h exposure to X-ray, all cells were harvested and washed with cold Phosphate Buffered Saline (PBS), and then fixed with 70% ethanol at 4 °C overnight. Before detecting, all samples were washed with 1× PBS, then incubated with 6 ul of 1 g/l RNase A, 1 ml of 1 mg/ml Propidium iodide (PI), and 400 ul of PBS at room temperature and protected from light for 15 min. Deoxyribonucleic acid (DNA) content and cell cycle distribution were analyzed using flow cytometry (FCM, BD FACS Calibur). All experiments were repeated for three times.

### Apoptosis analysis

TE-13 and Kyse150 cells were seeded in six-well plates (1 × 10^5^ per well) for 12 h and divided into the four groups: control, raltitrexed (4 nM), irradiation (8 Gy), and irradiation with raltitrexed (4 nM + 8 Gy). All cells were harvested and washed with cold PBS after 24 h exposure to raltitrexed followed by 24 h exposure to X-ray. Apoptotic cells were distinguished by Annexin V-FITC/PI dual staining using apoptosis detection kit from Keygen Biotech (Nanjing, China) and analyzed by flow cytometry (FCM, BD FACS Calibur). All experiments were repeated for three times.

### Immunofluorescence staining for phosphor-Histone H2AX (γ-H2AX) detection

DNA double strand breaks (DSBs) and DSB repair capacity were determined using immunofluorescence staining for γ-H2AX foci. TE-13 and Kyse150 cells were seeded on cover slips and then treated with either raltitrexed or DMSO (control). After 24 h of incubation, the cells were irradiated with 6 Gy of γ-rays and followed with 4% paraformaldehyde for 20 min at 2, 4, 24 h after irradiation. And then samples were permeabilized in 0.1% TritonX-100 for 10 min at 4 °C followed by washing with PBS and block with 5% bovine serum albumin at 37 °C for 1 h. Antibody against γ-H2AX (1:250) and 0.25 mg/ml DAPI (Beyotime, Jiangsu, China) were added for 5 min. Images were captured by a charge coupled device camera. For each group, γ-H2AX foci were counted in at least 50 cells per filed [14, 15]. Experiments were repeated for three times.

### Western blot analysis

TE-13 and Kyse150 cells were cultured and divided into eight groups: the control group, raltitrexed (4 nM) group, irradiation (4 Gy) groups (2 h, 4 h, 24 h) and raltitrexed combined with irradiation groups (2 h, 4 h, 24 h). Cells were collected and lysed with RIPA lysis buffer (Beyotime Biotechnology, Jiangsu, China). Protein concentrations were determined using BCA Protein Quantification Kit (Beyotime Technology, Shanghai, China). An equal amount of protein (50 ug) for each sample was resolved using 5% or 10% sodium dodecyl sulfate–polyacrylamide gel electrophoresis (SDS/PAGE) with MOPs running buffer and electrophoretically transferred onto a polyvinylidene difluoride (PVDF) membranes (IPVH00010, Millipore, Massachusetts, USA). The membrane was blocked with 5% bovine serum albumin at 37 °C for 1 h and sequentially blotted with primary antibodies (rabbit polyclonal or monoclonal antibodies to human Cyclin B1, Cdc25C, phosphor-Cdc25C^S216^ [pCdC25C], Cdc2, phospho-Cdc2^Thr14/Tyr15^[pCdc2], P53, P21^Waf1/Cip1^, γ-H2AX, Bax, c-Caspase-3, and folylpolyglutamate synthase [FPGS] 1:1000) and GAPDH antibody (1:2000), After washing with cold PBS, peroxidase-conjugated goat anti Rabbit IgG (1:5000) secondary antibody was added and followed by ECL detection. Experiments were repeated for three times.

### Xenograft tumor radiosensitivity studies

Animal experiments protocol was approved by Ethics Committee of Nanjing Medical University. Five to six week-old male BALB/C nude mice (Nanjing OGpharma technology lt. co) were injected with the suspension of 5 × 10^6^ TE-13 cells in 0.1 ml PBS into the right sub axillary. After 1–2 weeks post-injection, twenty-four nude mice with established tumors (all about 90 mm^3^) were divided into four groups: control group treated with (a) vehicle (PBS) alone; (b) Raltitrexed alone (7.5 mg/kg/day, continuous infusion at day 0–4 and day 7–11); (c) a single dose of 8 Gy IR; or (d) Raltitrexed plus IR (a single fraction of 8 Gy IR delivered on day 0 after Raltitrexed administration). Body weight and tumor diameter were measured every other day, and tumor volume was calculated according to the formula: (length [L] × width [W]^2^)/2. The tumor volume was recorded before and after treatment to evaluate the relative tumor volume (RTV) by Vt/V0 (Vt represents tumor volume, V0 represents pretreatment tumor volume). And the relative tumor control rate (T/C (%)) was determined by following formula: (RTV of Treatment group/ RTV of control group) × 100. After 19 days observation, tumors were harvested, fixed in 10% formalin, embedded in paraffin (FFPE), and mounted onto glass slides with 5 μm-thick sections for immunohistochemistry (IHC) assay.

### Immunohistochemistry

Slides were deparaffinized in xylene followed by rehydration in graded ethanol, rinsing twice with PBS, endogenous peroxidase blocking in 3% hydrogen peroxide for 15 min. Then the specimens were incubated with monoclonal antibody to PCNA (1:200, Abcam, Ltd., Cambridge, United Kingdom) or Ki-67 (1:200, Cell Signaling, Beverly, MA, USA) overnight at 4 °C and detected with horseradish peroxidase (HRP)-conjugated anti-rabbit secondary antibody for 1 h at room temperature or overnight at 4 °C. Next, the slides were visualized by incubation with 3,3-diaminobenzidine (DAB) (Dako, Hamburg, Germany), counterstained with hematoxylin (37%) and photographed using a Zeiss Axiovert A1 light microscope.

### Statistical analysis

All statistical analysis was performed using the GraphPad Prism software package version 6.0. All data were collected from three independent experiments and expressed as the mean ± standard deviation (SD). The Student’s t test or one-factor ANOVA was used to explore significant statistical differences between groups. P value less than 0.05 (p < 0.05) was considered statistically significant.

## Results

### Raltitrexed increased radiation sensitivity of ESCC cell lines

**Fig. 1:**
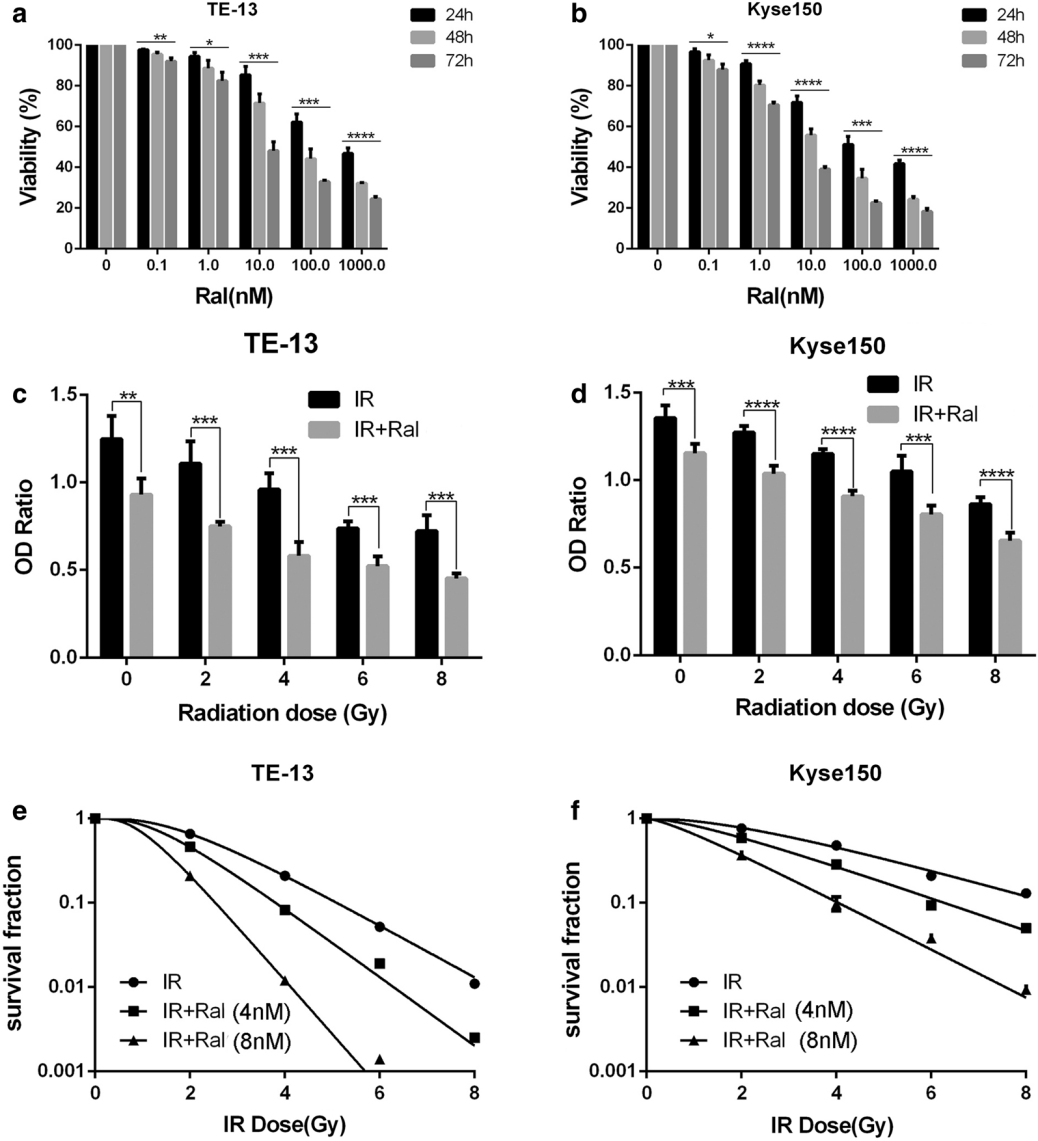
Raltitrexed (Ral) inhibited cell viability of esophageal squamous cancer cell lines and potentiated the inhibitory effect on cell viability and colony formation by irradiation (IR). The effects of different doses of Ral on the cell viability 24, 48 or 72 h’ treatment were determined in TE-13 (**a**) and Kyse150 cell lines (**b**); the effects of IR in different doses with or without Ral (4 nM) pretreatment (24 h) on the proliferation of TE-13 (**c**) and Kyse150 cell lines (**d**) were studied after 48 h using Cell counting kit 8 assay; the radiosensitization effect of Ral (4 nM, 8 nM) was studied in TE-13 (**e**) and Kyse150 cell lines (**f**) using clonogenic survival assay. Error bar: standard deviation; *p < 0.05, **p < 0.01, ***p < 0.001, ****p < 0.0001

TE-13 and Kyse150 cells were incubated with Raltitrexed at different concentrations for 24, 48 and 72 h. Raltitrexed inhibited the cell viability of TE-13 (Fig. 1a) and Kyse150 (Fig. 1b) cell lines in a time- and dose-dependent manner. Raltitrexed showed inhibitory effects on the growth of TE-13 and Kyse150 cells at concentrations ranging from 0.1 to 1000 nM. The half maximal inhibitory concentrations (IC50) of raltitrexed at 24 h in TE-13 and Kyse150 were 569.7 nM and 215.8 nM, respectively. The IC50 of raltitrexed at 72 h in TE-13 and Kyse150 were 19.61 nM and 6.19 nM, respectively (Table 1). Therefore, for the purpose of radiosensitivity, the low and safe concentrations of raltitrexed (4.8 nM) for 24 h treatment were applied in the following study. We choose these concentrations as these concentrations minimally inhibit the viability of the tumor cells.

**Table 1:** IC50 of Raltitrexed on TE-13 and Kyse150 for 24, 48 and 72 h

Cell line	IC50 (N = 5, mean ± SD, nmol/l)		
	24 h	48 h	72 h
TE-13	569.70 ± 20.41	94.76 ± 1.39	19.61 ± 0.43
Kyse150	215.80 ± 10.11	25.96 ± 1.93	6.19 ± 0.29

*SD* standard deviation

After exposure to raltitrexed at 4 nM for 24 h, the cells were subsequently treated with irradiation at different doses (0, 2, 4, 6, 8 Gy). 48 h later, cell proliferation capacity was evaluated. Raltitrexed (4 nM) combined with irradiation had better inhibitory effect than irradiation alone at different radiation doses, in either TE-13 (Fig. 1c) or Kyse150 cell line (Fig. 1d). The radiosensitizing effects of raltitrexed were also measured using colony forming assay. The colony numbers clearly decreased after combining raltitrexed with radiotherapy, compared with radiotherapy treatment alone (Fig. 1e, f). Survival fractions were fitted with single-hit multi-target model to estimate sensitizer enhancement ratio (SER). In TE-13 cells, the SER increased from 1.31 to 2.10 when the dose of raltitrexed given from 4 to 8 ng/l, while in Kyse150 cell line, the SER increased from 1.23 to 1.81. The sensitizer enhancement ratio (SER) and other radiobiological parameters of raltitrexed in TE-13 and Kyse150 cells are shown in Table 2. All the data demonstrated that raltitrexed increased cell death and suppression of cell proliferation along with irradiation in a dose dependent manner.

**Table 2:** Radio sensitization effect of raltitrexed on ESCC cells in vitro

	D_0_	Dq	N	SF (2 Gy)	SER
*TE-13*					
IR	1.40	0.83	3.92	0.66	
IR + Ral (4 nm)	1.07	0.61	3.71	0.46	1.31
IR + Ral (8 nm)	0.68	0.43	4.34	0.21	2.10
*Kyse150*					
IR	2.75	0.98	2.27	0.77	
IR + Ral (4 nm)	2.23	0.52	1.71	0.59	1.23
IR + Ral (8 nm)	1.52	0.25	1.45	1.10	1.81

*D*_*0*_ final slope, *Dq* quasi-threshold, *IR* irradiation, *nm* nmol/l, *Ral* raltitrexed, *SER* survival enhancement ratio, *SF* surviving fraction

### Raltitrexed promotes radiation-induced cell cycle distribution and protein expression alteration in TE-13 and Kyse150 cell lines

**Fig. 2:**
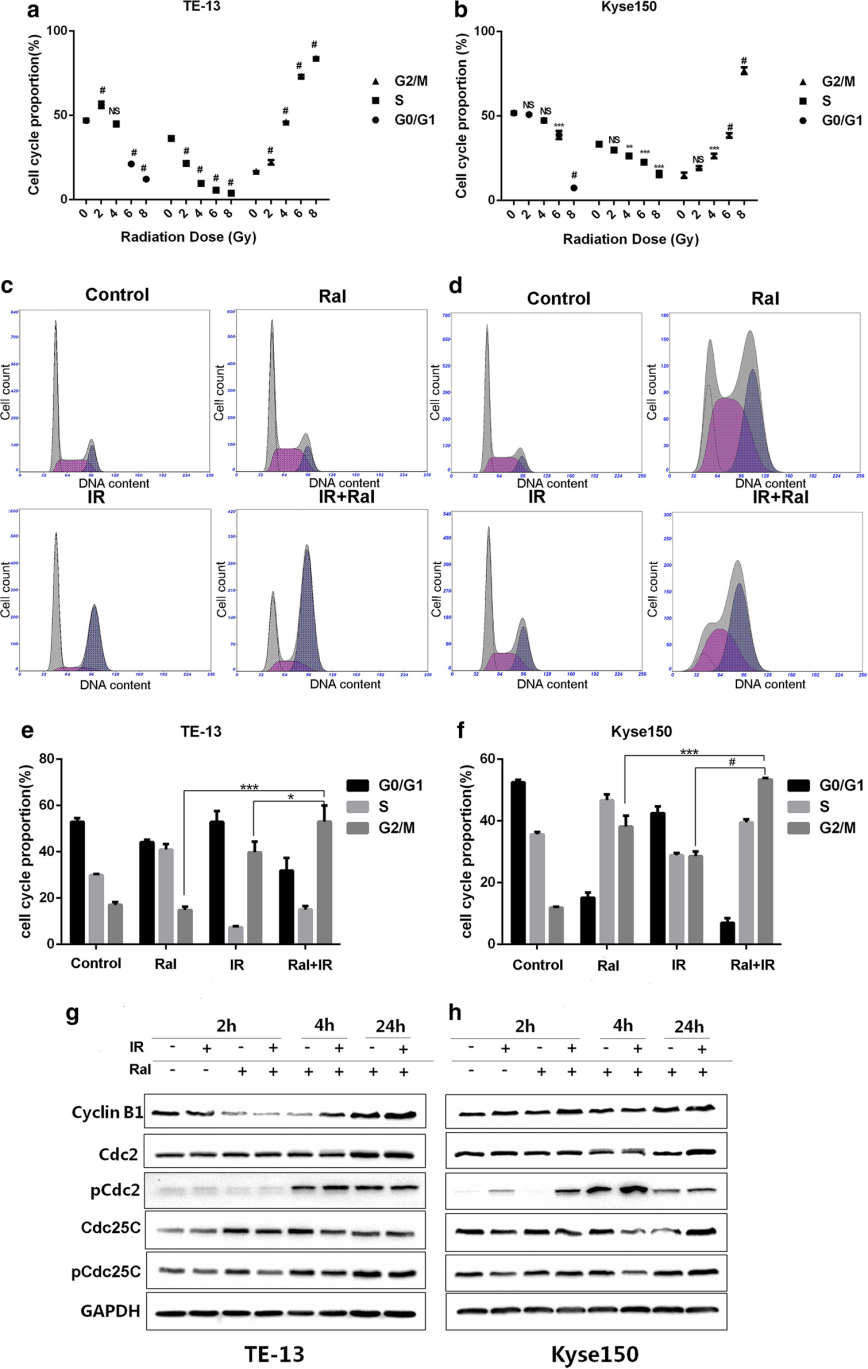
Raltitrexed (Ral) promoted irradiation (IR) induced cell cycle distribution and protein expression of TE-13 and Kyse150 cell lines. The effect of different doses of IR on cell cycle distribution in TE-13 (**a**) and Kyse150 cell lines (**b**); the effects of IR (4 Gy) with or without Ral (4 nM) pretreatment (24 h) on cell cycle were studied in TE-13 (**c**, **e**) and Kyse150 (**d**, **f**) cell lines 24 h after IR; the protein expression related to G2/M arrest in TE-13 (**g**) and Kyse150 (**h**) cell lines. Error bar: standard deviation; *p < 0.05, ***p < 0.001, ^#^p < 0.0001

To further understand the function of raltitrexed combined with irradiation in the ESCC cell lines, we detected the cell cycle distribution by flow cytometric analysis. Radiation alone induced G2/M arrest of TE-13 (Fig. 2a) and Kyse150 (Fig. 2b) cell lines. The G2/M arrest of the two cell lines increased in a dose dependent manner with radiation. The distribution of TE-13 and Kyse150 cells in the four different phases of cell cycle was analyzed after raltitrexed (4 nM) treatment for 24 h followed by radiation exposure (4 Gy) for 24 h (Fig. 2c, d). The percentages of cells in each phase among different groups were summarized in Fig. 2e, f. In both cell lines, G2/M arrest in the group of raltitrexed combined with irradiation was significantly increased compared with the radiation alone group and the raltitrexed alone group. As we know, DNA damage often induces G2/M phase arrest [16, 17] and Cdc2/Cyclin B1 complex is critical for regulating G2 to M transition. Western blot analysis (Fig. 2g) showed that pCdc2 (Thr14/Tyr15) was increased after treatment at different time points in TE-13 and Kyse150 cells. In Kyse150 cells, an earlier and more significant increase of pCdc2 was observed in raltitrexed combined with irradiation group, compared to irradiation alone group. The expression of Cyclin B1 was consistently with pCdc2, which was consistent with a G2 phase arrest. There are three Cdc25s in human cells, Cdc25A, Cdc25B and Cdc25C, and Cdc25C plays a central role in G2/M transition. At the beginning of cell mitosis, Cdc25C is activated and modulates Cdc2/Cyclin B1 complex. The expression of Cdc25c and pCdc25c (Ser216) were obviously increased at 24 h after treatment, which may indicate the beginning of mitosis.

### Raltitrexed increases radiation-induced apoptosis of TE-13 and Kyse150 cell lines

**Fig. 3:**
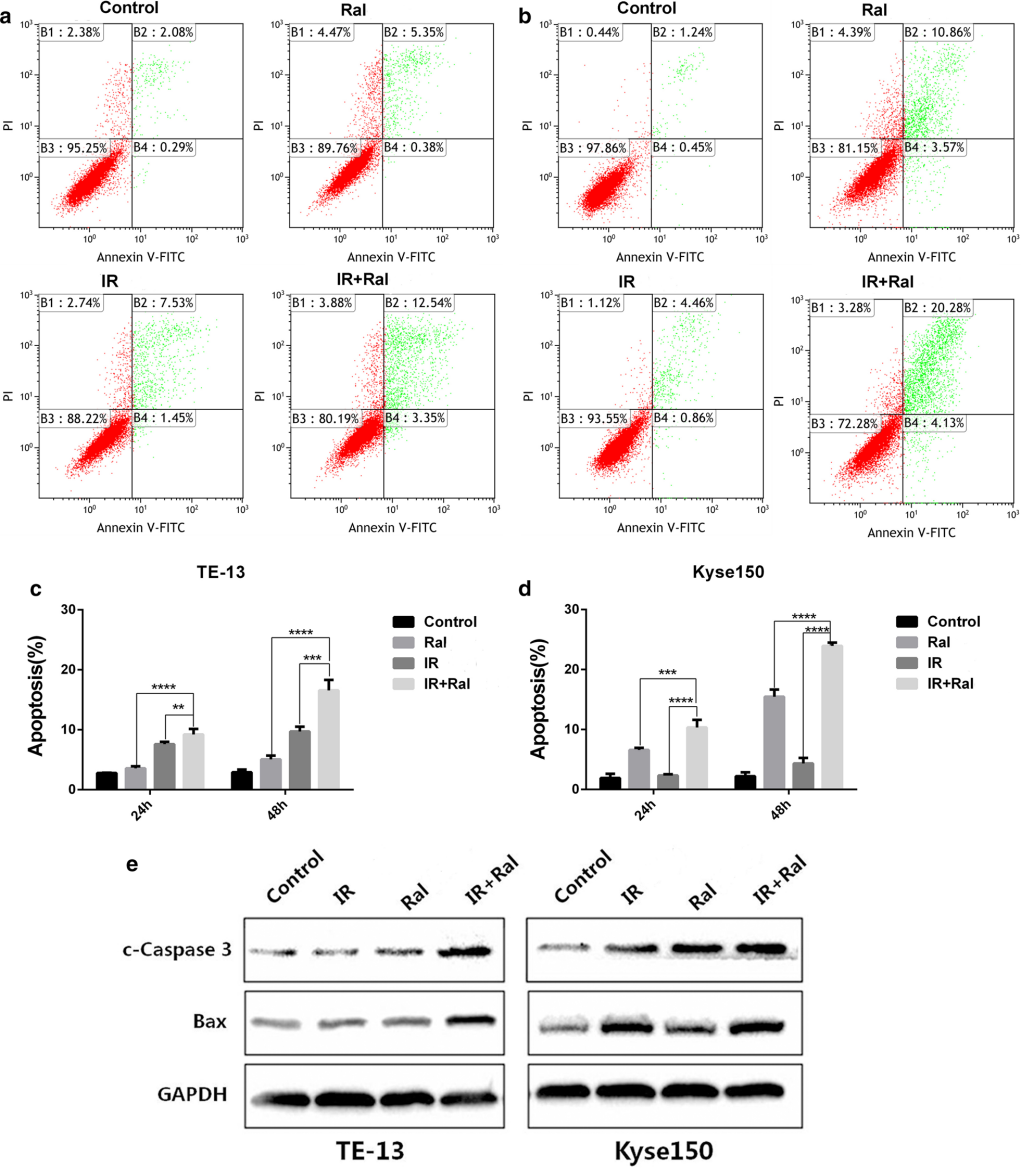
Raltitrexed (Ral) increased cell apoptosis in esophageal squamous cancer cells. The percentages of apoptosis cells were measured by flow cytometry after Ral (4 nM) treatment, irradiation (IR, 8 Gy, 24 h or 48 h) or combination of IR (8 Gy, 24 h or 48 h) + Ral (4 nM) in TE-13 (**a**, **c**) and Kyse150 (**b**, **d**) cell lines. Expression of apoptosis related proteins after different treatments was studied in TE-13 and Kyse150 (**e**). *p < 0.05, ***p < 0.001, ****p < 0.0001

The rates of apoptotic cells and dead cells were analyzed with or without raltitrexed pretreatment for 24 h at 24 h or 48 h after radiation exposure. The group of raltitrexed combined with irradiation (24 h and 48 h) showed a significantly higher apoptosis rate than the group of irradiations alone or the drug alone group in both TE-13 (Fig. 3a, c) and Kyse150 (Fig. 3b, d) cell lines. The proteins related to apoptosis were examined by western blot. The results indicated that c-Caspase 3 were remarkably increased in the combined group for TE-13 and Kyse150 cell lines compared to the irradiation alone group or the drug alone group (Fig. 3e). And pro-apoptosis protein Bax was also significantly elevated in the combination group when compared to the irradiation group or the drug alone group (Fig. 3e).

### Raltitrexed enhances radio-induced the formation of γ-H2AX nuclear foci of TE-13 and Kyse150 cell lines

**Fig. 4:**
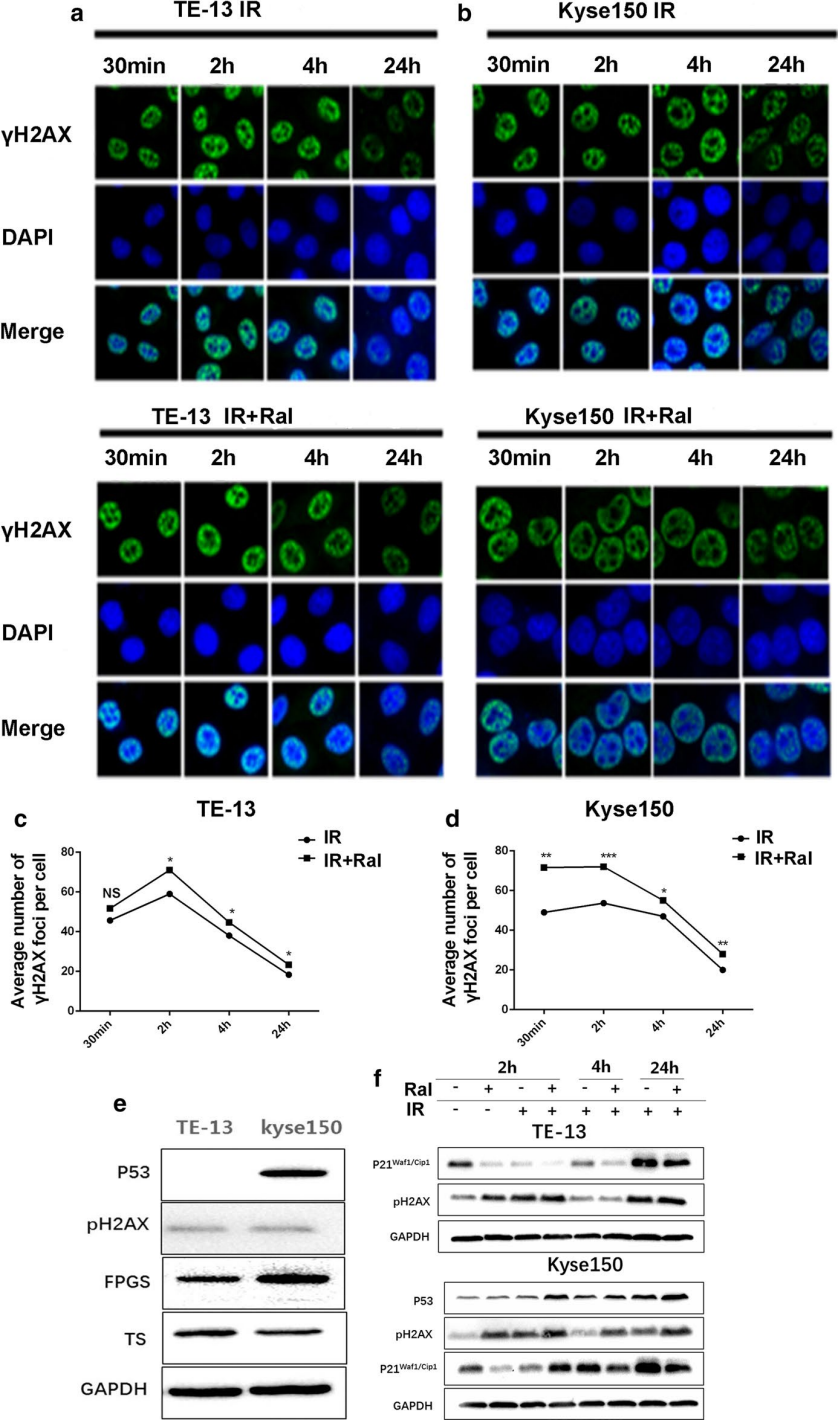
Raltitrexed (Ral) enhanced irradiation (IR) induced DNA damage in TE-13 and Kyse150 cell lines. γ-H2AX staining with Ral (4 nM) or without Ral (4 nM) pretreatment (24 h) in TE-13 (**a**, **c**) and Kyse150 (**b**, **d**) cell lines at each time point after IR exposure; TE-13 and Kyse150 cells were incubated with 0.1% DMSO or 4 nM Raltitrexed for 24 h and then exposed to 6 Gy of X ray for 2 h, 4 h or 24 h followed by immunofluorescence staining of γ-H2AX (green). Immunoreactive foci were ≥ 10 in nuclei that considered as positive for γ-H2AX. **e** Protein expression of P53, γ-H2AX, FPGS and TS in TE-13 and Kyse150 cells; **f** the effect of Ral (4 nM), IR (6 Gy) or combination of Ral (4 nM) and IR (6 Gy) in TE-13 and Kyse150 cells. *p < 0.05, **p < 0.01, ***p < 0.001

The impairment of DNA double-strand breaks (DSBs) repair could be a possible reason for raltitrexed-induced radiosensitization in cancer cells. Therefore, we evaluated DSBs levels through immunofluorescence staining of γ-H2AX foci in both TE-13 and Kyse150 cells at different time points after exposure to raltitrexed and/or X-rays. A higher number of γ-H2AX foci were observed at 2 h after exposure to X-rays, the majority of which were cleared at 4 h after exposure to 6 Gy of X-ray (Fig. 4a–d). As shown in Fig. 4e, we detected the protein expression of P53, phosphor-H2AX, FPGS, TS in TE-13 and Kyse150 cells. The results showed that P53 was deficient in TE-13 cells and was positive in Kyse150 cells While no obvious difference of phosphor-H2AX was seen between TE-13 and Kyse150 cells. Although P21 is regulated by P53, P21 was significantly upregulated after 24 h in the radiation alone group and the combination group. FPGS, which catalyzes the ATP-dependent addition of glutamate moieties to folate and folate derivatives, was less expressed in TE-13 while TS was more expressed in it compared to Kyse150 cells. Moreover, the average number of γ-H2AX foci subsided to near basal level at 24 h time point. The combination group always exhibited more γ-H2AX foci than the radiation alone group. The protein expression of γ-H2AX also certified this phenomenon (Fig. 4f). These data suggest that raltitrexed may increase radiosensitivity by inhibiting the repair of DSBs.

### Raltitrexed enhances radiation sensitivity in xenograft nude mice model

**Fig. 5:**
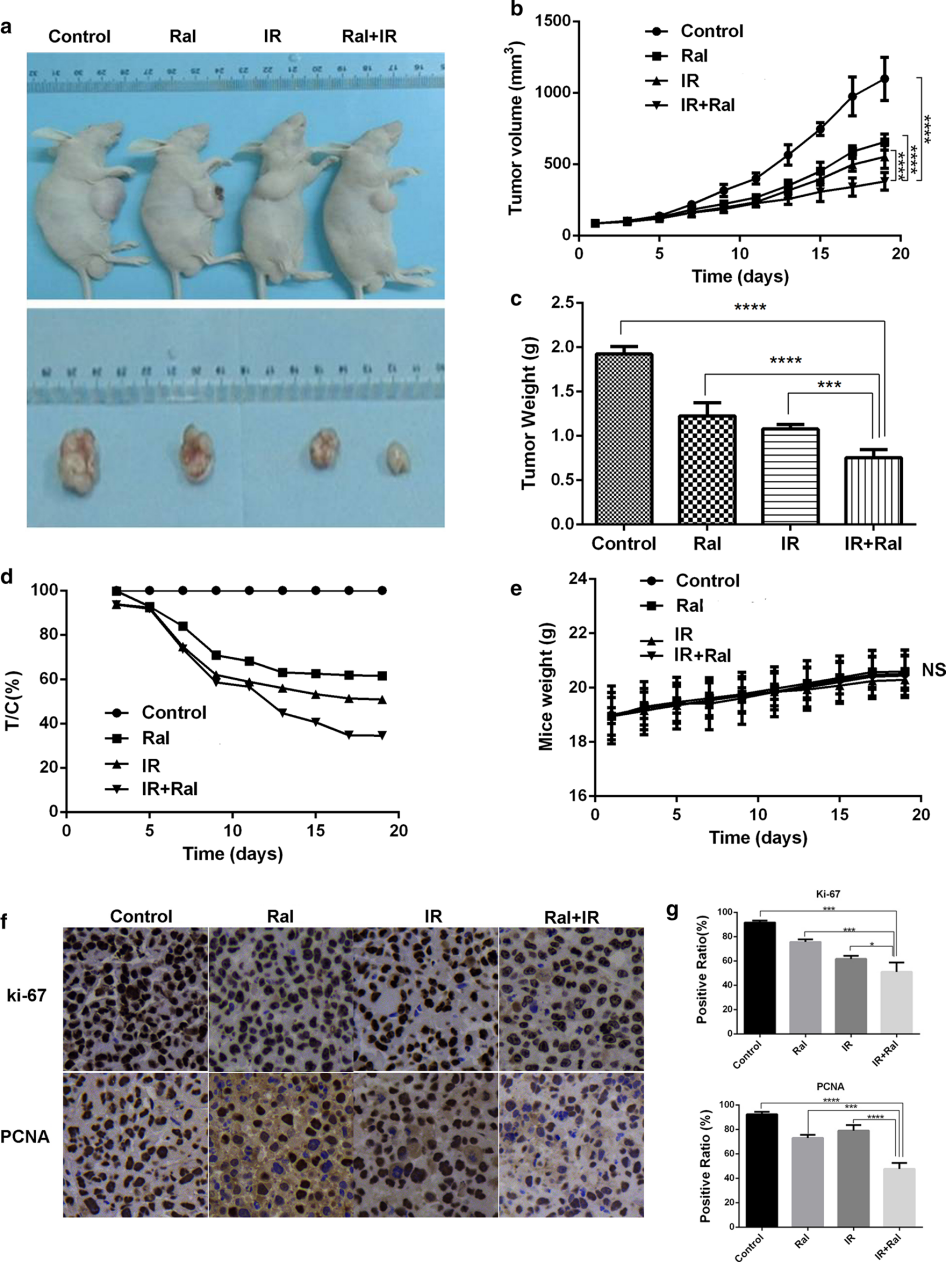
Raltitrexed (Ral) sensitized radiation (IR) induced anticancer effect in TE-13 xenografts. **a** The sizes of xenograft tumors in four different groups, including the control group, Ral group (7.5 mg/kg/day, day 0–4 and day 7–11), IR group (8 Gy day 0), and combination group; **b** tumor volumes in four groups; **c** T/C(%), tumor volume of treatment Group (T) compared with the control group (C); **d** mice weight in the four groups during the treatment; **e** comparison of tumor weight of the four groups; **f**, **g** immunohistochemistry of xenograft tumor for Ki-67 and PCNA. Error bar: standard deviation; *p < 0.05, ***p < 0.001, ****p < 0.0001

In order to confirm the potential radiosensitization effect of raltitrexed on ESCC tumor in vivo, TE-13 tumor xenograft mice model was established, which was treated with a single fraction of 8 Gy irradiation after raltitrexed administration on day 0. And then mice were given intraperitoneal injection of raltitrexed (0.75 mg/kg/day) for 0–4 and 7–11 days. Table 3 showed that raltitrexed inhibited the proliferation of TE-13 xenograft combined with Radiation. There is no distinct difference of tumor volume was observed between raltitrexed-treated groups and irradiation group. Compared to control group, significant reduction of tumor volume was observed in the treatment group. Tumor volume and tumor weight of the group treated with both irradiation and raltitrexed were significantly decreased compared with the group treated with irradiation or raltitrexed alone (p < 0.05) (Fig. 5a–d). No obvious weight loss was observed among all groups, which indicated treatments were well tolerated by the nude mice (Fig. 5e). Immunohistochemical analysis demonstrated that raltitrexed inhibited Ki-67 and PCNA expression in either irradiation treated or untreated tumors (Fig. 5f). The mean density of Ki-67 and PCNA staining in tumors from raltitrexed treated groups was significantly lower than control group. These data indicated that raltitrexed radiosensitized ESCC in vivo.

**Table 3:** Raltitrexed inhibits the proliferation of TE-13 xenograft combined with radiation

Groups	TV (mm^3^)	Inhibitory rate of tumor volume (%)	Mice weight (g)	Inhibitory rate of tumor weight (%)
Control	12.91 ± 1.562	–	1.923 ± 0.085	–
Raltitrexed	7.945 ± 1.770*	38.5	1.225 ± 0.150*	36.3
Irradiation	6.575 ± 1.396*	49.1	1.078 ± 0.05*	43.9
IR + Ral	4.458 ± 0.528*	65.5	0.755 ± 0.093*	60.7

*IR* irradiation, *Ral* raltitrexed, *TV* tumor volume

*p < 0.05

## Discussion

Our study demonstrated that raltitrexed could enhance the radiosensitivity of ESCC cells in vitro and in vivo. The viability and proliferation of ESCC cells were decreased in a time and dose-dependent manner by raltitrexed incubation. And the apoptosis was increased by raltitrexed combined with irradiation. Furthermore, we found G2/M phase arrest contributed to radiosensitization of raltitrexed through the upregulation of Cdc2/Cyclin B1 complex and the increased expression of γ-H2AX foci, which indicated the inhibition of DNA-damage repair. Besides, we observed that raltitrexed sensitized TE-13 xenograft tumor to irradiation. Therefore, raltitrexed could be used as a radiosensitizer to treat ESCC.

To prevent cells from entering into a new phase, a number of cell cycle checkpoints have been made to ensure proper cell cycle progression. It is widely accepted that the cell cycle phase determined different degrees of radiosensitivity of cells to radiation. G2 and M phases are the critical time points that cells are most sensitive to irradiation and most radioresistant time point was S phase cell cycle. Cdc2/Cyclin B1 complex activity has been considered as an import role in G2/M phase transition. Several studies have indicated that G2/M cell cycle arrest showed decreased the expression of Cdc2/Cyclin B1 [18, 19]. Increased Cdc2/Cyclin B1 activity can promote radiation-induced cell apoptosis by inducing G2/M transition [20, 21]. However, cyclin B1 is also related to radioresistance [22]. In this work, we detected the changes in the cell cycle progression by using flow cytometry and western blot. The results showed that radiation alone induced G2/M-phase arrest in TE-13 and Kyse150 cells, while combined with raltitrexed, the G2/M arrest was significantly increased. Meanwhile, the protein expression of Cyclin B1, pCdc2, pCdc25c were increased after exposing to raltitrexed combined with irradiation. These results may be related to the transition from G2 phase to M phase. G1/S and G2/M transition are the two major checkpoints of cell cycle, on the occasion of DNA damage or incomplete DNA replication, the checkpoints can prevent inappropriate cell cycle progression [23]. The G2/M checkpoint is known to involve a number of proteins, including P53, P21, Cyclin B1, Cdc2, Cdc25C, Chk1 and so on [24, 25]. P53 is a tumor suppressor, which can induce cell apoptosis, cell cycle arrest and DNA repair in response to a variety of intrinsic or extrinsic factors [26]. In certain situations, P53 may actually lead damaged cells to self-destruct in order to prevent damaged genetic goods handed down [26, 27]. Reportedly, P53 is required for P21 induction following exposure to radiation [28] and P21 can be regulated independently of P53 in certain situations. In our study, we found that the protein expression of P53 was positive in Kyse150 cells and was obviously increased after treatment, especially in the case of raltitrexed combined with Radiation, while in TE-13 cells, P53 was negative and we also observed the increased expression of P21 at 24 h after treatment, obviously later than in Kyse150 cells. The increased P53 and P21 can inhibit DNA damaged cells from entering into mitosis. At the beginning of cell mitosis, Cdc25C is activated and modulates Cdc2/Cyclin B1 complex. The complex of Cdc2/Cyclin B1 at G2/M transition keeps an inactive state by phosphorylation of Cdc2 at Thr-14 and Tyr-15. Our results found that in both TE-13 and Kyse150 cell lines, Cdc2 was remarkably phosphorylated at 4 h after irradiation treatment, especially higher in the combined group, which indicated G2 checkpoint arrest. Moreover, the expression of pCdc2 decreased quickly at 24 h exposure in Kyse150 cell line, which suggested that cells were unable to sustain stable G2 arrest. Meanwhile, the activation of Cdc25C may result in the start of mitosis for cells, which initiates cell death program due to the uncompleted DNA repair. In the TE-13 cell line, this process may be delayed, which can partly explained why the apoptosis rate in TE-13 cells was less than that in Kyse150 cells at 24 h after treatment and was more than in Kyse150 cells at 48 h after treatment.

Raltitrexed has been considered as a specific inhibitor of TS [29] and could predominantly enter the cell through the reduced folate carrier (RFC) and then undergo polyglutamation by FPGS enzyme. The polyglutamated compound stayed in cells and acted as an anticancer agent. Previous studies have found that raltitrexed could inhibit HepG2 cell proliferation via G0/G1 arrest [7], inducing the apoptosis of cancer cells [6]. And we observed that raltitrexed could increase radiation-induced G2/M arrest and apoptosis in ESCC cells, and the latter was confirmed by the increase of c-caspase-3 expression. C-caspase-3 has been identified as an executer of apoptosis that was associated with two signaling pathways, including the mitochondrial and the cell death receptor pathway. And the activation of Caspase-3 is essential for DNA fragmentation. Therefore, the expression of c-Caspase-3 was measured to show the cytotoxic responsiveness as an index. Based on our results, we conclude that apoptosis induction was a possible mechanism for increased radiosensitivity of ESCC cells that was induced by raltitrexed. In this study, we also observed an increasing expression of phosphor-H2AX after raltitrexed treatment in TE-13 and Kyse150 cells, which suggested increased DNA damage. As we known, DNA DSBs are associated with cell cycle arrest and/or death due to unrepaired molecular lesions [30]. Cell death may involve in a single unrepaired or misrepaired DNA DSB of a functional gene section [31]. A previous study suggested that raltitrexed treatment in HCT-8 cells resulted in DNA fragmentation and was accompanied with elevated P53 protein expression [32]. In our study, we found elevated P53 accompanied with overexpression of γ-H2AX in Kyse150 cell line.

Above all, our study demonstrated for the first time that raltitrexed could significantly enhance the radiosensitivity of ESCC cells via inducing G2/M arrest by activating the Cdc2/Cyclin B1 pathway and increasing cell apoptosis. Moreover, raltitrexed could sensitize TE-13 xenograft tumor to irradiation. These results suggested that raltitrexed could be a potent radiosensitization agent for ESCC treatment in the future. In this study, we also observed different protein expression of P53 in TE-13 and Kyse150 cells, showing that P53 was negative in TE-13 while Kyse150 was positive. There are probably other mechanisms involved in the radiosensitization effects of raltitrexed in ESCC cell lines, which needs further investigation.

## Conclusion

Raltitrexed enhances the radiosensitivity of ESCC in vitro and in vivo, therefore can be used with concurrent radiation in esophageal cancer in clinical trials.
